# Tenovin-6 impairs autophagy by inhibiting autophagic flux

**DOI:** 10.1038/cddis.2017.25

**Published:** 2017-02-09

**Authors:** Hongfeng Yuan, Brandon Tan, Shou-Jiang Gao

**Affiliations:** 1 Department of Molecular Microbiology and Immunology, Keck School of Medicine, University of Southern California, Los Angeles, CA, USA

## Abstract

Tenovin-6 has attracted significant interest because it activates p53 and inhibits sirtuins. It has anti-neoplastic effects on multiple hematopoietic malignancies and solid tumors in both *in vitro* and *in vivo* studies. Tenovin-6 was recently shown to impair the autophagy pathway in chronic lymphocytic leukemia cells and pediatric soft tissue sarcoma cells. However, whether tenovin-6 has a general inhibitory effect on autophagy and whether there is any involvement with SIRT1 and p53, both of which are regulators of the autophagy pathway, remain unclear. In this study, we have demonstrated that tenovin-6 increases microtubule-associated protein 1 light chain 3 (LC3-II) level in diverse cell types in a time- and dose-dependent manner. Mechanistically, the increase of LC3-II by tenovin-6 is caused by inhibition of the classical autophagy pathway via impairing lysosomal function without affecting the fusion between autophagosomes and lysosomes. Furthermore, we have revealed that tenovin-6 activation of p53 is cell type dependent, and tenovin-6 inhibition of autophagy is not dependent on its regulatory functions on p53 and SIRT1. Our results have shown that tenovin-6 is a potent autophagy inhibitor, and raised the precaution in interpreting results where tenovin-6 is used as an inhibitor of SIRT1.

Tenovin-6 is a monocarboxylic acid amide with the formal chemical name '*N*-[[[4-[[5-(dimethylamino)-1-oxopentyl] amino] phenyl] amino] thioxomethyl]-4-(1, 1-dimethylethyl)-benzamide'. Tenovin-6 has attracted significant interest because it was originally identified as a potent p53 activator, and shown to have direct inhibitory effect on the protein deacetylase activity of purified human SIRT1, SIRT2 and SIRT3 *in vitro* with IC50 values of 21, 10 and 67 *μ*M, respectively.^1, 2^ Since then, tenovin-6 has been widely used as an inhibitor of sirtuins, especially SIRT1, and demonstrated to have a promising anti-neoplastic effect *in vitro* or *in vivo* on hematopoietic malignancies including chronic myelogenous leukemia,^3, 4, 5, 6^ chronic lymphocytic leukemia (CLL),^7, 8^ acute lymphoblastic leukemia,^9^ acute promyelocytic leukemia^10^ and solid tumors such as pancreatic cancer,^11^ colon cancer,^12^ gastric cancer,^13^ Ewing sarcoma,^14^ soft tissue sarcoma^15^ and uveal melanoma.^16^ Tenovin-6 was used at doses (1–10 *μ*M) much lower than the IC50 values for sirtuins in most of these studies; however, it still strongly induced cell arrest or apoptosis, suggesting that it may have a function other than inhibiting sirtuins.

Tenovin-6 has recently been shown to impair the autophagy pathway by increasing the levels of LC3-II (lipidated form of LC3) and/or SQSTM1/p62 in CLL cells^7, 8^ and pediatric soft tissue sarcoma cells.^15^ However, it is unclear whether tenovin-6 has a general effect on autophagy on other types of cells. Furthermore, the mechanism by which tenovin-6 inhibits autophagy, particularly whether SIRT1 and p53, two of the crucial regulators of autophagy pathway, are involved remains unknown.

In this study, we have found that tenovin-6 increases LC3-II level in all types of cells tested in a time- and dose-dependent manner. The increase of LC3-II by tenovin-6 results in the inhibition of classical autophagy pathway by impairing lysosomal function without affecting the fusion between autophagosome and lysosome. Furthermore, we have revealed that tenovin-6 activation of p53 is cell type dependent, and both p53 and SIRT1 are not involved in tenovin-6 inhibition of the autophagy pathway. Our results have identified tenovin-6 as a potent autophagy inhibitor, and raised the precaution in interpreting data when tenovin-6 is used as an inhibitor of SIRT1.

## Results

### Tenovin-6 increases the level of LC3-II in diverse cell types

**Figure 1:**
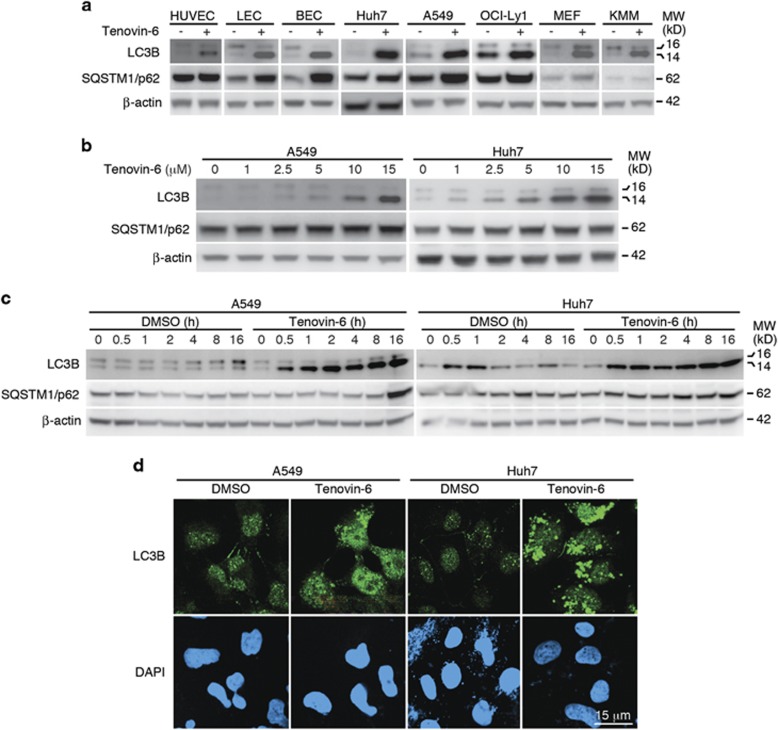
LC3-II is increased following tenovin-6 treatment. (**a**) A panel of different types of cells was treated with tenovin-6 for 24 h. LC3B, SQSTM1/p62 and *β*-actin were examined by western blotting. Tenovin-6 was used at 5* μ*M except for A549 cells where 10* μ*M was used. (**b**) A549 and Huh7 cells were treated with indicated concentrations of tenovin-6 for 24 h. LC3B, SQSTM1/p62 and *β*-actin were examined by western blotting. (**c**) A549 and Huh7 cells were treated with 10 or 5* μ*M tenovin-6, respectively, for the indicated times. LC3B, SQSTM1/p62 and *β*-actin were examined by western blotting. (**d**) A549 and Huh7 cells were treated with 10 and 5 *μ*M tenovin-6, respectively, for 16 h, and examined for LC3B by immunofluorescence assay

To determine whether tenovin-6 has a general effect on autophagy, we examined the lipidated form of LC3, LC3-II, following treatment with tenovin-6 by western blotting in a panel of different types of cells including mouse embryonic fibroblasts (MEFs), primary human umbilical vein endothelial cells (HUVEC), primary human lymphatic endothelial cells (LEC), primary human bone marrow endothelial cells (BEC), human hepatocarcinoma cell line Huh7, human lung adenocarcinoma epithelial cell line A549, diffuse large B-cell lymphoma cell line (DLBCL) OCI-Ly1, rat embryonic metanephric mesenchymal cells transformed by Kaposi's sarcoma-associated herpesvirus (KMM).^17^ LC3-II consisting of LC3A-II and LC3B-II are the hallmark products of autophagy. The LC3B-II level was markedly increased after treatment with tenovin-6 for 24 h in all types of cells examined, whereas LC3A-II was increased in most cell types examined except Huh7 and OCI-Ly1 cells, which had relatively low expression levels of LC3A-II (Supplementary Figure S1). The specificity of the antibody against LC3A or LC3B was confirmed with Huh7 cells following overexpression of a mCherry-tagged LC3A or mCherry-tagged LC3B (Supplementary Figure S2). The increase of LC3-II products suggested that tenovin-6 affected autophagy in these cells. We further examined the dose dependence and time kinetics of LC3B-II increase after tenovin-6 treatment. Treatment of A549 and Huh7 cells with tenovin-6 from 0 to 15 *μ*M for 24 h showed that LC3B-II increase was dose dependent (Figure 1b). LC3B-II started to increase at as early as 0.5 h when these cells were treated with 10 *μ*M of tenovin-6 in A549 cells or 5 *μ*M of tenovin-6 in Huh7 cells for up to 24 h (Figure 1c).

As the increase of LC3B-II can occur during either induction or inhibition of autophagy, we further examined the p62 level to clarify the effect of tenovin-6 on autophagy. The induction of autophagy often leads to degradation of SQSTM1/p62.^18^ Surprisingly, the expression level of SQSTM1/p62 was either unchanged or increased in different types of cells examined following tenovin-6 treatment despite the increase of LC3-II (Figure 1a), suggesting that the autophagic flux was inhibited. There was also no reduction of SQSTM1/p62 in A549 and Huh7 cells treated with different doses of tenovin-6 for 24 h (Figure 1b), and with 10 or 5 *μ*M tenovin-6 for different lengths of time, respectively (Figure 1c).

To confirm the results of western blotting, we performed confocal microscopy examination of LC3B following immunofluorescence staining with a LC3B antibody. Treatment with 10 or 5 *μ*M of tenovin-6 for 16 h increased the signal of LC3B and the typical puncta structures in A549 and Huh7 cells, respectively (Figure 1d).

Taken together, these results indicated that tenovin-6 treatment increased LC3-II level in diverse types of cells; however, the autophagic flux was blocked.

### The increase of LC3-II after tenovin-6 treatment is ATG5/7 dependent

**Figure 2:**
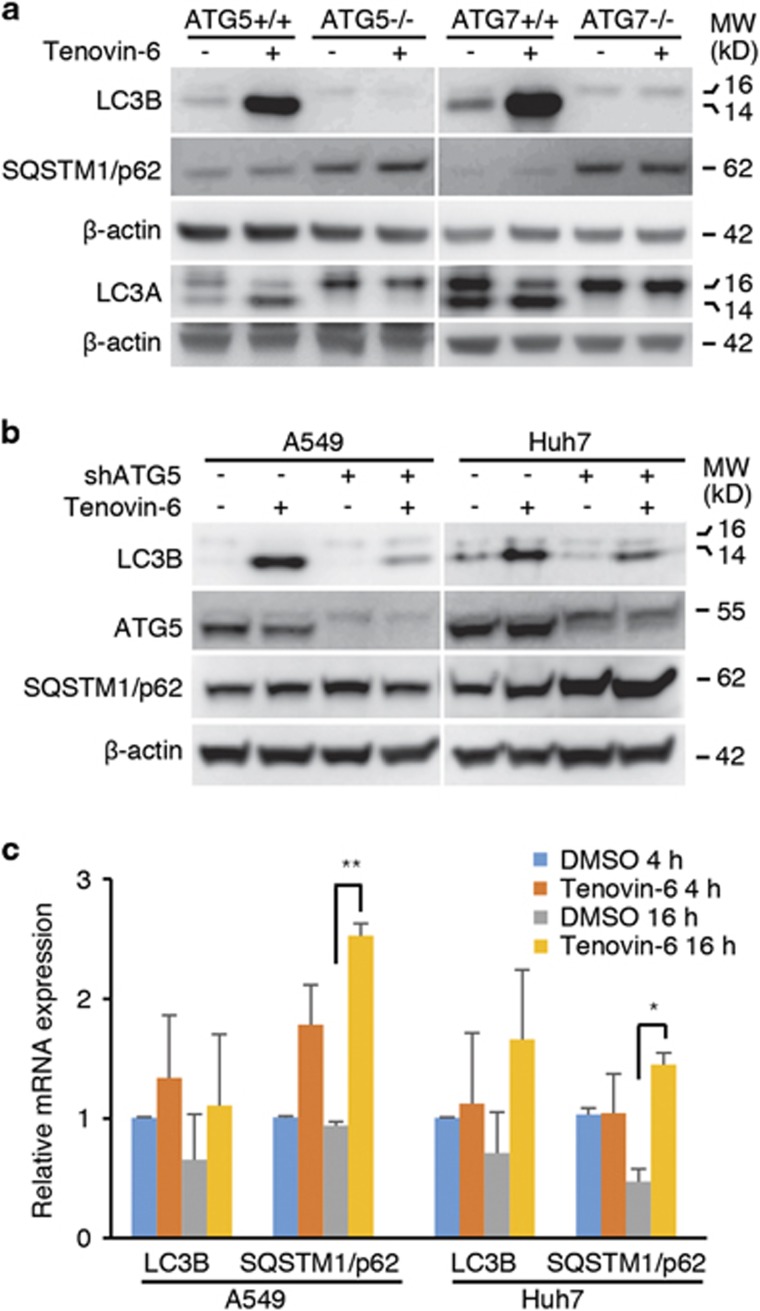
The increase of LC3-II by tenovin-6 is ATG5/7 dependent. (**a**) LC3B, LC3A, SQSTM1/p62 and *β*-actin were examined by western blotting in ATG5/7 wild-type and knockout MEF cells after treatment with 5 *μ*M tenovin-6 for 24 h. (**b**) At day 4 post-infection with shRNA lentiviruses specific for ATG5 (shATG5), A549 and Huh7 cells were treated with 10 and 5 *μ*M of tenovin-6, respectively, for another 8 h. LC3B, ATG5 and *β*-actin were then examined by western blotting. (**c**) The mRNA levels of LC3B and SQSTM1/p62 in A549 and Huh7 cells were examined by RT-qPCR following treatment with 10 and 5 *μ*M of tenovin-6, respectively, for 4 h and 16 h. *P<0.05, **P<0.01

As both ATG5 and ATG7 are essential for LC3-II conversion in the classical autophagy pathway, we took advantage of the ATG5^−/−^ and ATG7^−/−^ MEF cells to examine their roles in the increase of LC3-II by tenovin-6. In the ATG5/7-deficient MEF cells, LC3-II formation is blocked.^19, 20, 21^ We found that tenovin-6 treatment significantly increased LC3A/B-II in wild-type but not ATG5^−/−^ and ATG7^−/−^ MEF cells (Figure 2a). Examination of SQSTM1/p62 showed that there was no change in SQSTM1/p62 level in either wild-type or ATG5^−/−^ and ATG7^−/−^ MEF cells following tenovin-6 treatment. The increase of LC3B-II following tenovin-6 treatment was also markedly compromised in human A549 and Huh7 cells following knockdown of ATG5 (Figure 2b). These results indicated that tenovin-6 inhibited autophagy mediated by the canonical ATG5/7 activity. Although LC3B-II is an excellent marker for autophagy, its level could be regulated at both transcriptional and post-translational levels. To exclude the possibility that the increase of LC3-II level following tenovin-6 treatment was not due to transcriptional upregulation, we examined the mRNA level of LC3B in A549 and Huh7 cells after tenovin-6 treatment for 4 and 16 h by quantitative reverse transcription real-time PCR (RT-qPCR). We found that the mRNA level of LC3B was largely unchanged following tenovin-6 treatment (Figure 2c), indicating that transcriptional regulation was not the mechanism accounting for the marked protein increase of LC3-II following tenovin-6 treatment. For comparison, the mRNA level of SQSTM1/p62 was only slightly increased in A549 and Huh7 cells at 16 h after tenovin-6 treatment (Figure 2c). Taken together, our results indicated that the increase of LC3-II after tenovin-6 treatment was ATG5/7 dependent.

### Tenovin-6 prevents Torin 1-induced SQSTM1/p62 degradation

**Figure 3:**
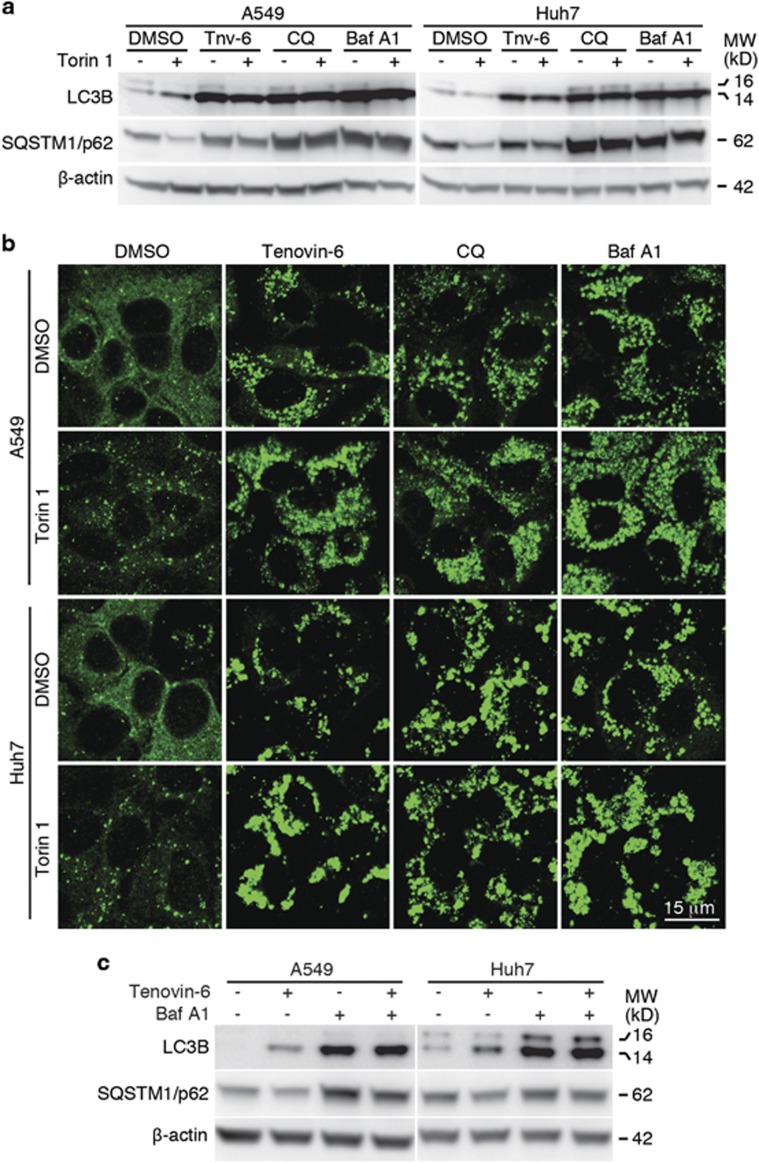
Tenovin-6 prevents Torin 1-induced SQSTM1/p62 degradation. (**a**) LC3B, SQSTM1/p62 and *β*-actin were examined by western blotting following the indicated treatments for 16 h. The concentrations of the compounds used were 10* μ*M tenovin-6 (Tnv-6), 100 *μ*M chloroquine (CQ), 25 nM bafilomycin A1 (Baf A1) and 250 nM Torin 1 for A549 cells; and 5 *μ*M Tnv-6, 50* μ*M CQ, 25 nM Baf A1 and 250 nM Torin 1 for Huh7 cells. (**b**) SQSTM1/p62 was examined by immunofluorescence assay following the indicated treatments for 16 h. The concentrations of the compounds used were the same as **a**. (**c**) LC3B, SQSTM1/p62 and *β*-actin were examined by western blotting after the indicated treatments for 8 h. Tenovin-6 was used at 10 *μ*M for A549 and 5 *μ*M for Huh7, whereas Baf A1 was used at 25 nM for all cells

The increase of LC3-II could be attributed to either the induction of early stages of autophagy or the inhibition of late stages of autophagic flux. However, as we did not detect reduced expression of SQSTM1/p62 (Figures 1a–c, Figures 2a and b), tenovin-6 likely inhibited late stages of autophagic flux. To further confirm the inhibitory effect of tenovin-6 on autophagic flux, we challenged the cells with Torin 1, a potent inhibitor of catalytic mechanistic target of rapamycin, to induce autophagy and examined SQSTM1/p62 level by western blotting and immunofluorescence assay. Torin 1 treatment induced LC3B-II (Figure 3a). Chloroquine or bafilomycin A1, two known inhibitors of autophagic flux, also increased the level of LC3B-II as tenovin-6 did. As expected, Torin 1 treatment induced SQSTM1/p62 degradation (Figure 3a). Tenovin-6 not only increased SQSTM1/p62 level but completely blocked Torin 1-induced SQSTM1/p62 degradation in both A549 and Huh7 cells. Similar results were also observed with chloroquine or bafilomycin A1. By staining for SQSTM1/p62 with a specific antibody in an immunofluorescence assay, we further confirmed these results (Figure 3b). Torin 1 treatment clearly induced autophagic vesicles in both A549 and Huh7 cells. Tenovin-6 treatment increased the number and intensity of autophagic vesicles with or without the presence of Torin 1. Taken together, these results indicated that tenovin-6 inhibited the autophagic flux induced by Torin 1.

To further show the inhibitory effect of tenovin-6 on autophagic flux, we treated A549 and Huh7 cells with tenovin-6 in the presence of a known autophagic flux inhibitor bafilomycin A1, and examined the effect on LC3B and SQSTM1/p62. If tenovin-6 is an inducer of the autophagic flux, the combined treatment of tenovin-6 with a saturated inhibitor like bafilomycin A1 should lead to an additive effect on LC3B expression. However, if tenovin-6 is an inhibitor of the autophagic flux, there should not be any additive effects because bafilomycin A1 should have completely blocked the autophagic flux. We chose 25 nM bafilomycin A1 for the treatment as it was confirmed to be saturated in both A549 and Huh7 cells (Supplementary Figure S3). Combined treatment with both tenovin-6 and bafilomycin A1 did not have any significant additive effect, indicating that tenovin-6 was indeed an inhibitor of the autophagic flux (Figure 3c).

### Tenovin-6 does not affect the fusion between autophagosomes and lysosomes

**Figure 4:**
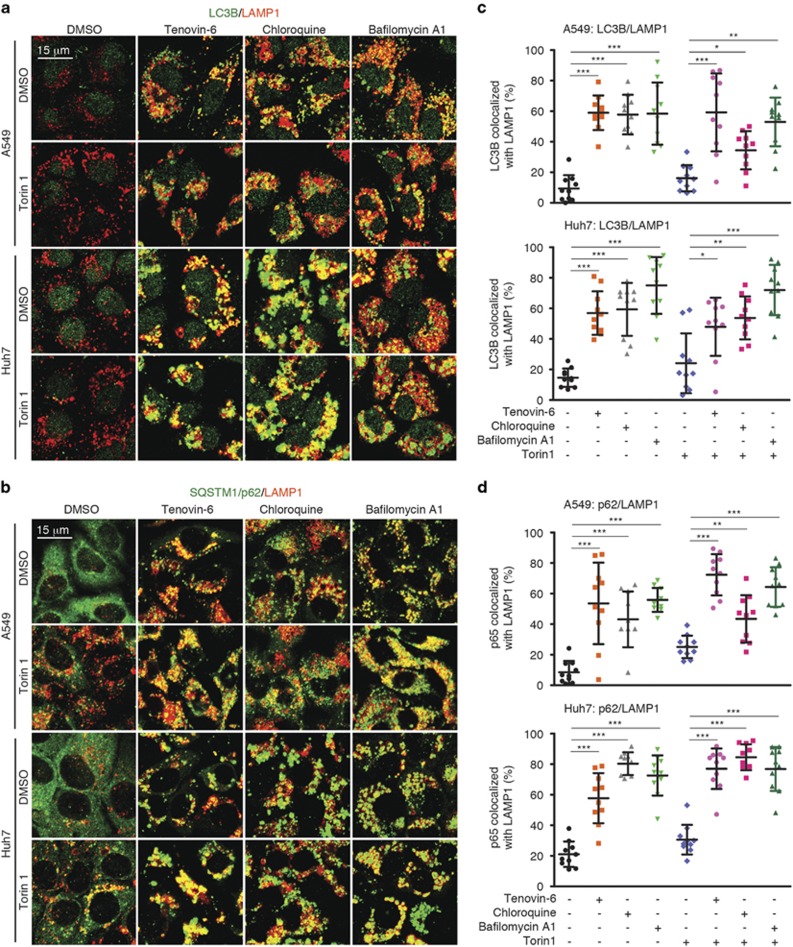
Tenovin-6 does not affect the fusion between autophagosome and lysosome. (**a** and **b**) Colocalization of LC3B with LAMP1 (**a**) and SQSTM1/p62 with LAMP1 (**b**) were examined by immunofluorescence assay following the treatment with the indicated agents for 16 h. The concentrations of compounds used were 10* μ*M tenovin-6, 100 *μ*M chloroquine, 25 nM bafilomycin A1 and 250 nM Torin 1 for A549 cells; and 5 *μ*M tenovin-6, 50* μ*M chloroquine, 25 nM bafilomycin A1 and 250 nM Torin 1 for Huh7 cells. (**c** and **d**) The percentages of overlapping fluorescence of LC3B with LAMP1 (**c**) and SQSTM1/p62 with LAMP1 (**d**) were analyzed by fluorescence intensity profiling using the NIS-Elements 4.5 Software. Overlapping fluorescence was defined by (the number of overlapping peaks)/(total green fluorescence peaks). *P<0.05, **P<0.01, ***P<0.001

To identify the specific autophagy stage targeted by tenovin-6, we examined the fusion between autophagosomes and lysosomes by co-staining endogenous autophagosome marker LC3B and lysosome marker LAMP1 (lysosome-associated membrane protein 1) in A549 and Huh7 cells. We detected co-staining of both LC3B and LAMP1, and enlarged autophagic vesicles in both cells at as early as 6 h following tenovin-6 treatment (Supplementary Figure S4). At later time points, we observed more marked co-staining of both LC3B and LAMP1, and enlarged autophagic vesicles in both A549 and Huh7 cells treated with tenovin-6, chloroquine or bafilomycin A1 (Figure 4a). LC3B-positive autolysosomes decorated by a well-defined LAMP1-positive ring were clearly visible (Figure 4a). Furthermore, the colocalization of endogenous SQSTM1/p62 with LAMP1 was obvious after treatment with tenovin-6, chloroquine or bafilomycin A1 (Figure 4b). On the other hand, there were few autophagic vesicles with dual staining of LC3B and LAMP1 (Figure 4a), as well as SQSTM1/p62 and LAMP1 (Figure 4b) after Torin 1 treatment, indicating fast autophagic flux and rapid degradation of LC3B and SQSTM1/p62 once transition to lysosome. We further used the NIS-Elements 4.5 Software to quantify the LC3B or SQSTM1/p62 protein signal that co-stained with LAMP1 signal by drawing a total of 10 random lines for each image (Supplementary Figure S4). As many as 59–60% and 58–78% of LC3B vesicles (autophagic vesicles) in A549 and Huh7 cells, respectively, had overlapping fluorescence signal with LAMP1 signal following treatment with tenovin-6, chloroquine or bafilomycin A1 compared with only 10% and 16% of LC3B vesicles that had overlapping fluorescence signal with LAMP1 signal in the untreated cells, respectively (Figure 4c). Similarly, as much as 45–57% and 59–80% of SQSTM1/p62 in A549 and Huh7 cells, respectively, had overlapping fluorescence signal with LAMP1 signal following treatment with tenovin-6, chloroquine or bafilomycin A1 compared with only 9% and 21% of SQSTM1/p62 that had overlapping fluorescence signal with LAMP1 signal in the untreated cells, respectively (Figure 4d). Treatment with Torin 1 did not significantly alter the amount of LC3B or SQSTM1/p62 co-stained with LAMP1 in both A549 and Huh7 cells. The increase of co-staining signal of LC3B and SQSTM1/p62 with LAMP1 by tenovin-6, chloroquine or bafilomycin A1 was not altered by co-treatment with Torin 1, ranging from 37% to 60% and 44% to 74% for LC3B, respectively, and from 42% to 74% and 78% to 84% for SQSTM1/p62, respectively, in A549 and Huh7 cells (Figures 4c and d). These results indicated that tenovin-6, as well as chloroquine and bafilomycin A1 did not affect the fusion between autophagosomes and lysosomes.

### Tenovin-6 affects the acidification of autolysosomes and impairs the hydrolytic activity of lysosomes

**Figure 5:**
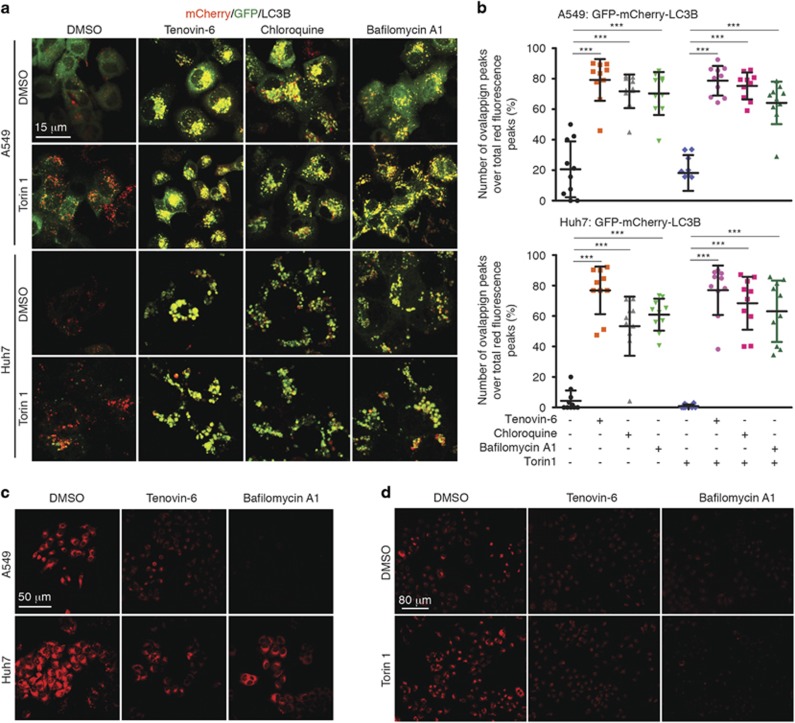
Tenovin-6 affects the acidification of autolysosomes and impairs the hydrolytic activity of lysosomes. (**a**) Colocalization of mCherry and GFP in live stable mCherry-GFP-LC3B-expressing cells following the indicated treatments. A549 cells were treated with 10 *μ*M tenovin-6, 100 *μ*M chloroquine, 25 nM bafilomycin A1 and 250 nM Torin 1 for 4 h. Huh7 cells were treated with 5 *μ*M tenovin-6, 50* μ*M chloroquine, 25 nM bafilomycin A1 and 250 nM Torin 1 for 8 h. (**b**) The percentage of overlapping fluorescence analyzed by fluorescence intensity profiling using the NIS-Elements 4.5 Software. Overlapping fluorescence was defined by (the number of overlapping peaks)/(total red fluorescence peaks). ***P<0.001. (**c**) LysoTracker staining in cells following the indicated treatments. A549 cells were treated with 10* μ*M tenovin-6 and 25 nM bafilomycin A1 for 2 h. Huh7 cells were treated with 5* μ*M tenovin-6 and 25 nM bafilomycin A1 for 2 h. (**d**) Magic Red staining following the treatment of A549 cells with 10* μ*M tenovin-6 or 25 nM bafilomycin A1 for 3 h in the presence or absence of 250 nM Torin 1

To test the acidification of autophagic vesicles in the live cells after tenovin-6 treatment, we used the tandem-tagged mCherry-GFP-LC3B as a dual-fluorescence pH sensor. This fusion protein emits both GFP and mCherry signals in the pH neutral autophagosomes; however, the GFP but not mCherry fluorescence signal is quenched upon autophagic delivery to the acidic environment in the lysosomes. Therefore, both red and green fluorescence signals are present in autophagosomes, whereas only the red fluorophore is visible in acidic autolysosomes.^22^ We established stable cultures of mCherry-GFP-LC3B in both A549 and Huh7 cells. Most of autophagic vesicles had only red fluorescence signal with few having both red and green signals in cells treated with Torin 1 (Figure 5a). However, close to 80% of the autophagic vesicles had strong dual-fluorescence signals following treatment with tenovin-6 alone or together with Torin 1 (Figure 5b). Similar results were also observed with chloroquine and bafilomycin A1 (Figures 5a and b). The GFP fluorescence signal was clearly maintained in almost all autophagic vesicles in cells treated with tenovin-6, chloroquine or bafilomycin A1 regardless of the presence or absence of Torin 1. As our results had already shown that tenovin-6, chloroquine or bafilomycin A1 did not affect the fusion between autophagosomes and lysosomes (Figure 4), these results strongly suggested that the acidification of autolysosomes was impaired by tenovin-6, chloroquine and bafilomycin A1.

To further confirm that the acidification of lysosomes/autolysosomes was affected by tenovin-6, we used LysoTracker to monitor the change of pH. LysoTracker is a cell-permeable pH-dependent dye that accumulates in acidic vesicles such as lysosomes or autolysosomes in live cells and emits fluorescence signal. LysoTracker staining has been commonly used to monitor the pH in the lysosome/autolysosome.^23, 24, 25, 26^ Although both A549 and Huh7 cells had strong signal following LysoTracker staining, treatment with tenovin-6 or bafilomycin A1 for 2 h significantly reduced the signal, thus confirming that tenovin-6 affected the acidification of lysosomes/autolysosomes (Figure 5c).

To test whether tenovin-6 affected the acidification-dependent hydrolytic function of lysosomes/autolysosomes, we used the Magic Red cathepsin B reagent CV-(RR)_2_ (Cresyl Violet-(Arg-Arg)_2_) to detect the activity of cathepsin B. Cathepsin B is one of the major cysteine proteases primarily activated in acidic lysosomes/autolysosomes. CV-(RR)_2_ does not emit fluorescence signal. However, following enzymatic hydrolysis by cathepsin B, the two R-R peptide sequences are cleaved, releasing the Magic Red molecule and converting it to the red fluorescence form. We detected red fluorescence signal in A549 cells treated with CV-(RR)_2_ (Figure 5d). Treatment with tenovin-6 or bafilomycin A1 for 3 h significantly reduced the fluorescence signal, indicating inhibition of the cathepsin B activity by tenovin-6 or bafilomycin A1 (Figure 5d). It has recently been shown that Torin 1 can activate cathepsin B activity in the course of autophagy in MEF cells.^25^ Indeed, treatment with Torin 1 for 3 h markedly increased the fluorescence signal (Figure 5d). However, treatment with Torin 1 together with tenovin-6 or bafilomycin A1 completely abolished the enhancing effect of Torin 1 (Figure 5d). Thus, we concluded that the hydrolytic activity in the lysosomes/autolysosomes was impaired by tenovin-6.

### Autophagy inhibition by tenovin-6 does not correlate with p53 activation

**Figure 6:**
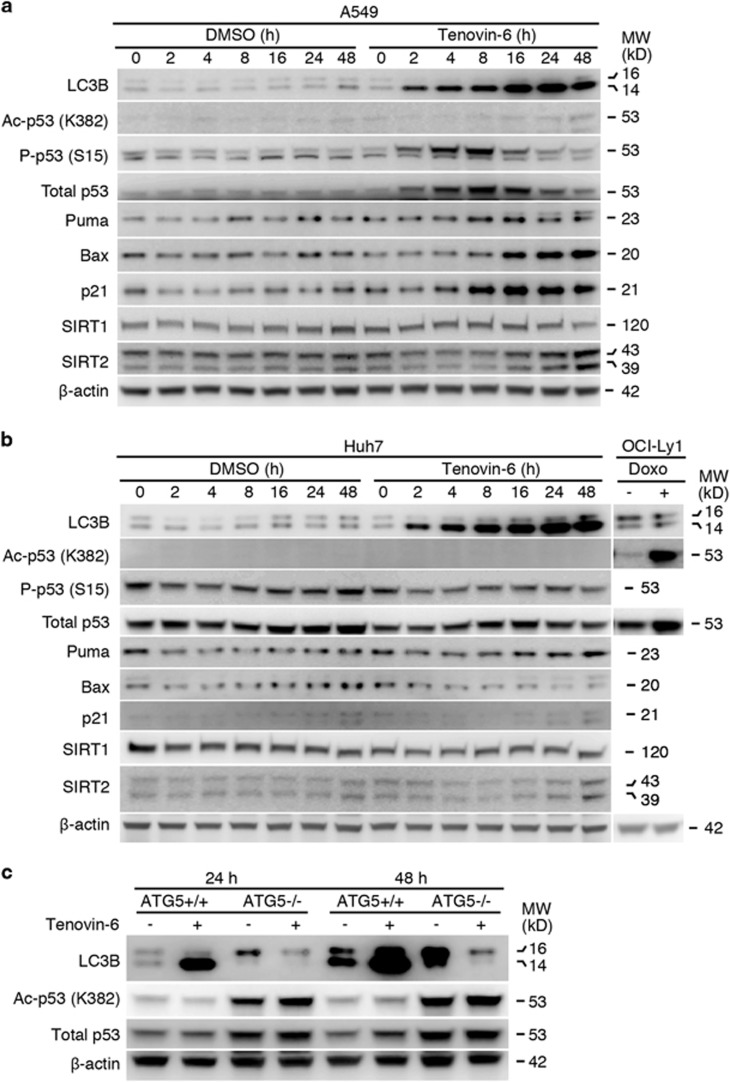
Inhibition of autophagy by tenovin-6 does not correlate with p53 activation. (**a** and **b**) Total p53 and its acetylation form at K382, its phosphorylation form at S15, SIRT1, SIRT2, Bax, Puma, p21, LC3B and *β*-actin in A549 (**a**) and Huh7 (**b**) cells following tenovin-6 treatment at the indicated time points examined by western blotting. Tenovin-6 was used at 10 *μ*M for A549 cells and 5* μ*M for Huh7 cell. OCI-Ly1 cells treated with 2 *μ*M doxorubicin (Doxo) for 7 h were used as a positive control for p53 acetylation. (**c**) Total p53 and its acetylation form at K382, LC3B and *β*-actin in ATG5 wild-type (ATG^+/+^) and knockout (ATG^−/−^) MEF cells were examined by western blotting after treatment with 5 *μ*M tenovin-6 for 24 and 48 h

Tenovin-6 was originally identified as a p53 activator in breast cancer cell line MCF-7 and lung cancer cell line H1299.^1^ Our results showed that tenovin-6 had a general effect on LC3B-II accumulation. As p53 regulates the autophagic pathway,^27, 28^ we simultaneously examined p53 activation and LC3B-II accumulation following tenovin-6 treatment. Although we detected LC3B-II accumulation in cells treated with tenovin-6 from 0 to 48 h, we failed to detect consistent p53 activation based on the status of p53 acetylation (K382) and phosphorylation (Ser15), and induction of its downstream targets Puma, Bax and p21 in all the time points examined (Figures 6a and b). p53 acetylation was clearly detected in OCI-Ly1 cells treated with doxorubicin (Figure 6b) but not in A549 and Huh7 cells following tenovin-6 treatment (Figures 6a and b). Interestingly, the total p53 level and its phosphorylation (Ser15) were increased at as early as 2 h following tenovin-6 treatment, and p53 downstream targets Bax and p21 were also induced correspondingly in A549 cells (p53 WT) (Figure 6a) but not in Huh7 cells (p53 mutant) (Figure 6b). Tenovin-6 had no effect on the p53 acetylation status in ATG5^−/−^ MEF cells after treatment for 24 h and 48 h, and in ATG5^+/+^ MEF cells after treatment for 24 h (Figure 6c). There were slight increases of total p53 level and its acetylation only at 48 h after tenovin-6 treatment in ATG5^+/+^ MEF cells. Loss of ATG5 (ATG5^−/−^) markedly increased p53 expression level and its acetylation status (K379 in mouse) (Figure 6c). These results indicated that the increase of LC3B-II by tenovin-6 did not correlate with p53 activation, which was in line with the observation in CLL^7^ and soft tissue sarcoma cells.^15^

### SIRT1/2 inhibition by knockdown or knockout cannot mimic the effect of tenovin-6 on LC3B accumulation

**Figure 7:**
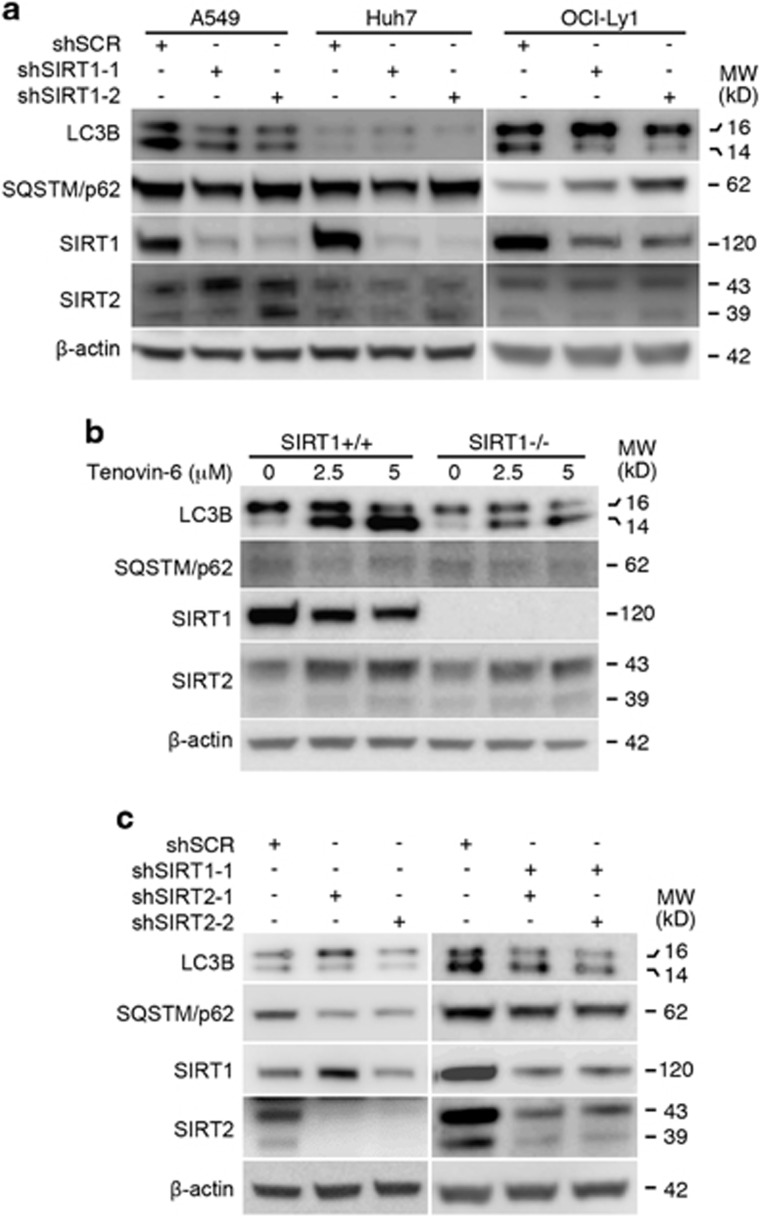
Inhibition of SIRT1/2 by knockdown or knockout does not cause LC3B-II accumulation. (**a**) LC3B, SQSTM1/p62, SIRT1, SIRT2 and *β*-actin were examined by western blotting in A549, Huh7 and OCI-Ly1 cells following SIRT1 knockdown. A549 and Huh7 cells were collected 4 days after transduction without selection, whereas OCI-Ly1 cells were selected in 2.5 *μ*g/ml puromycin for 5 days before collection. (**b**) LC3B, SQSTM1/p62, SIRT1, SIRT2 and *β*-actin were examined by western blotting in SIRT1 wild-type (SIRT^+/+^) and knockout (SIRT1^−/−^) KMM cells after treatment with 2.5 *μ*M and 5 *μ*M tenovin-6 for 24 h. (**c**) LC3B, SQSTM1/p62, SIRT1, SIRT2 and *β*-actin were examined by western blotting in OCI-Ly1 cells following SIRT2 knockdown or SIRT1/2 double knockdown. Cells were collected 4 days after transduction without selection

It was originally shown that tenovin-6 could directly inhibit the activity of NAD^+^-dependent protein deacetylase SIRT1. Tenovin-6 has since been used as an inhibitor of SIRT1 in a number of studies.^1, 3, 4, 5, 6, 9, 10, 11, 12, 13, 14, 15, 16^ As SIRT1 regulates the autophagic pathway,^29, 30, 31^ we examined whether knockdown of SIRT1 was sufficient to mimic the effect of tenovin-6 on LC3B-II accumulation. SIRT1 knockdown did not cause LC3B-II accumulation in A549, Huh7 and OCI-Ly1 cells; in contrast, it slightly reduced the LC3B-II levels (Figure 7a). There was slight increase of SQSTM1/p62 in OCI-Ly1 but not in A549 and Huh7 cells following SIRT1 knockdown. In KMM cells, treatment with tenovin-6 at 2.5 or 5 *μ*M caused LC3B-II accumulation (Figure 7b). SIRT1 knockout KMM cells (SIRT1^−/−^) generated using the Crispr/Cas9 system did not cause LC3B-II accumulation. The loss of SIRT1 actually resulted in the reduction of LC3B expression level and blocked the effect of tenovin-6 on LC3B-II accumulation (Figure 7b). Tenovin-6 has been shown to target both SIRT1 and SIRT2. Knockdown of SIRT1 may not be sufficient to mimic the effect of tenovin-6 in these cells because SIRT2 may compensate the loss of SIRT1. SIRT2 was expressed in A549, Huh7, OCI-Ly1 and KMM cells, and did not have any significant change following SIRT1 knockdown, or knockout (Figures 7a and b). Therefore, we generated SIRT1/SIRT2 double knockdown OCl-Ly1 cells and examined LC3B-II and SQSTM1/p62 levels. Again, SIRT1/SIRT2 double knockdown did not lead to LC3B-II increase (Figure 7c). These results indicated that the effect of tenovin-6 on LC3B-II accumulation cannot be phenocopied by knockdown of SIRT1 or SIRT2 alone, or double knockdown of both SIRT1 and SIRT2.

## Discussion

Autophagy is an evolutionarily conserved cellular homeostatic process in response to starvation or other stress conditions. During this process, proteins, organelles and intracellular pathogens are engulfed into autophagosomes and eventually delivered to lysosomes for degradation. A growing body of evidence has demonstrated a critical role of autophagy in the pathogenesis of diverse diseases, such as neuronal degeneration, pathogen infection, myopathy, aging and cancer. Results of extensive preclinical studies in tumor cells in culture and in animal models have suggested that autophagy induced by antitumor drugs and radiation is cytoprotective and can be targeted to overcome chemotherapeutic resistance. Clinical trials combining autophagy inhibitor such as hydroxychloroquine with other drugs have shown promising results in some cases.^32^ In this study, we have provided extensive evidences to show tenovin-6 as a general autophagy inhibitor with strong similarity to hydroxychloroquine or bafilomycin A1. As tenovin-6 has promising anti-neoplastic effect in culture or in animal models on hematopoietic malignancies and solid tumors, our results suggest that the underlying mechanisms for the anti-neoplastic effect of tenovin-6 could be through inhibition of autophagy apart from inhibition of sirtuins. Thus, combining tenovin-6 as an autophagy inhibitor with other drugs to overcome chemotherapeutic resistance could be a viable therapeutic approach for some types of cancer.

SIRT1 regulates a wide variety of physiological and pathological processes through deacetylating its substrates.^33^ Recent studies have identified a crucial role of SIRT1 in the induction of autophagy. On one hand, SIRT1 could directly deacetylate and activate the key components of the autophagy pathway including autophagy genes ATG5, ATG7 and LC3 (i.e., ATG8).^29, 31^ On the other hand, SIRT1 could induce the expression of autophagy pathway components through deacetylating and activating transcription factors of the FoxO family members.^30^ In this study, we have shown that SIRT1 knockdown or knockout reduces the expression of LC3B, results that are consistent with results of aforementioned studies. However, the results of SIRT1 knockdown or knockout are opposite to those of tenovin-6 treatment, which significantly increases LC3B accumulation. In a recent study, tenovin-6 treatment was shown to increase LC3-II accumulation, whereas siRNA knockdown of SIRT1 or SIRT2 decreased or did not change LC3-II level in pediatric soft tissue sarcoma cells.^15^ Hence, tenovin-6 induction of LC3-II accumulation is not through the pharmacological inhibition of SIRT1 and SIRT2 albeit the authors have paradoxically made such a conclusion.^15^ More recently, the acetylomes of SIRT1 wild-type or knockout MEF cells, as well as wild-type MEF cells treated with 10 *μ*M of tenovin-6 or DMSO as a control for 16 h were compared; however, no correlation was detected between SIRT1 knockout cells and cells treated with tenovin-6.^34^ We have recently shown that tenovin-6 increases accumulation of LC3B-II and inhibits the classical autophagy pathway in a SIRT1/2/3- and p53-independent manner in DLBCL cell lines.^35^ These results along with those of this study suggest that precaution should be taken to interpret the data where tenovin-6 is used as an inhibitor of SIRT1.

The tandem-tagged mCherry-GFP-LC3B is commonly used as a pH sensor in the live cells based on the fact that the fluorescence from GFP is acid sensitive and therefore is quenched in the acidic condition, whereas the fluorescence from mCherry is acid insensitive.^22^ In the autophagic condition with normal lysosomal function, the double-tagged LC3B can be used to distinguish autophagosomes from autolysosomes. In autophagosomes, both tags emit fluorescence signals, whereas in the acidic autolysosomes, the green fluorescence from GFP is lost. However, in the situation where lysosomal acidification cannot be well maintained, this protein cannot be used to separate autophagosomes from autolysosomes because the GFP signal remains visible in non-acidic autolysosomes. The formation of autolysosomes seems independent of acidification.^26^ In this situation, co-staining of endogenous autophagosome marker LC3 or SQSTM1/p62 with lysosome marker LAMP1 would be more accurate and straightforward to distinguish autophagosomes from autolysosomes as shown in this study. Using this strategy, we have found that autolysosomes are still formed after treatment with tenovin-6, chloroquine or bafilomycin A1. As for chloroquine, our results are consistent with published results, in which active fusion between autophagosomes and lysosomes, and the delivery of autophagic cargos to lysosomes are not affected after chloroquine treatment in MEF cells and multiple human cell lines.^24^ With regard to bafilomycin A1, the results from two other studies show that the formation of autolysosomes is blocked by bafilomycin A1 treatment, which contradicts with our observations.^26, 36^ In these studies, mCherry-tagged LC3B and GFP-tagged LAMP1 were used, which may not reflect the real situation in the cells. In contrast, we have directly stained endogenous LC3B or SQSTM1/p62 together with LAMP1 with their respective specific antibodies. The results have been confirmed with antibodies against endogenous LAMP1 from two different sources.

In summary, we have demonstrated that tenovin-6 causes LC3-II accumulation in diverse cell types and in a dose- and time-dependent manner. Mechanistically, the LC3-II accumulation is caused by inhibition of the classical autophagy pathway via impairing lysosomal function, and is p53- and SIRT1 independent. Our study has identified tenovin-6 as a potent autophagy inhibitor, and suggests that precaution should be taken to interpret the data where tenovin-6 is used as a SIRT1 inhibitor.

## Materials and Methods

### Reagents and antibodies

The following reagents from different the manufacturers were used in this study: tenovin-6 (Cat# BSCC-37, Agave Pharm, Palo Alto, CA, USA), chloroquine diphosphate (C6628, Sigma, St. Louis, MO, USA), bafilomycin A1 (B1793, Sigma), Torin 1 (4247, Tocris Bioscience, Bristol, UK), LysoTracker Red DND-99 (L7528, Invitrogen, Carlsbad, CA, USA), Magic Red cathepsin B reagent (#937, Immunochemistry Technologies, LLC, Bloomington, MN, USA) and doxorubicin (2252, Tocris Bioscience).

The antibodies used were: LC3B (#CTB-LC3-1-50, Cosmo Bio, San Diego, CA, USA), LC3A (#ab62720, Abcam, Cambridge, MA, USA), SQSTM1/p62 (#PM045, MBL, Woburn, MA, USA), ATG5 (#2630, CST, Danvers, MA, USA), LAMP1 (#ab25630, Abcam; #9091, CST), *β*-actin (#sc-8432, Santa Cruz, Santa Cruz, CA, USA), p53 (DO-1 for human and sc-126 for mouse, Santa Cruz; #2524, CST), acetyl-p53 (K382) (#2524, CST), acetyl-p53 (K379) (#2570, CST), phospho-p53 (S15) (#9284, CST), SIRT1 (#8469, CST), SIRT2 (#12672, CST), Bax (#2772, CST), Puma (#4976, CST), p21 (#556430, BD Biosciences, Franklin Lakes, NJ, USA), goat anti-rabbit IgG-HRP (#SC-2030, Santa Cruz) and goat anti-mouse IgG-HRP (#SC-2005, Santa Cruz).

### Cell culture

Immortalized MEFs ATG5^+/+^, ATG5^−/−^, ATG7^+/+^ and ATG7^−/−^ cells kindly provided by Dr. Chengyu Liang, rat embryonic metanephric mesenchymal cells transformed by Kaposi's sarcoma-associated herpesvirus (KMM),^17^ human embryonic kidney 293 cells HEK 293T and human hepatocarcinoma cell line Huh7 were cultured in Dulbecco’s modified Eagle’s medium. Human lung adenocarcinoma epithelial cell line A549 cells were cultured in RPMI-1640 media. Diffuse large B-cell lymphoma cell line OCI-Ly1 was cultured in Iscove's modified Dulbecco's medium. All these media are supplemented with 10% fetal bovine serum (26140-079, ThermoFisher Scientific, Waltham, MA, USA), 100* μ*g/ml penicillin and 100* μ*g/ml streptomycin. Primary HUVECs, primary LECs and primary BECs were cultured in VascuLife VEGF Complete Medium (LM-0024, Lifeline Cell Technology, Frederick, MD, USA). All cells were maintained in a 5% CO_2_ atmosphere at 37 °C.

### Lentiviral shRNA knockdown

The ATG5 shRNA (TRCN0000150940, 5′-GCAGAACCATACTATTTGCTT-3′) was purchased from Sigma. A scrambled shRNA (5′-TTGTACTACACAAAAGTACTG-3′) was constructed as a control. Two shRNAs targeting SIRT1 (#1: 5′-GAAGTGCCTCAGATATTAA-3′ and #2: 5′-GTTGACCTCCTCATTGTTA-3′) and two shRNAs targeting SIRT2 (#1: 5′-GCTTATTGGAGACAAATTA-3′ and #2: 5′-GAAACATCCGGAACCCTTC-3′) were constructed as previously described.^6^ The lentivirus was generated and produced as previously described.^6^ Cells were transduced with the lentivirus particles by spinning infection at 1800 r.p.m. for 60 min in the presence of 8* μ*g/ml polybrene. MOI 5 was used for A549 and Huh7, and MOI 12 was used for OCI-Ly1.

### Stable mCherry-GFP-LC3B-expressing cell cultures

The retroviral vector pBABE-puro-mCherry-EGFP-LC3B was obtained from Addgene (Cambridge, MA, USA, #22418). The retrovirus was generated as previously described.^6^ A549 and Huh7 cells were transduced by spinning infection at 1800 r.p.m. for 60 min in the presence of 8* μ*g/ml polybrene. At 48 h post-transduction, cells were selected in 2.5 *μ*g/ml puromycin for 3 weeks.

### Immunofluorescence assay

Cells of 5x10^4^ were plated on coverslips in 24-well plates and cultured for 24 h. Cells were treated as indicated and fixed with ice-cold methanol for 10 min at room temperature. After fixation, the cells were washed and blocked with 3% BSA in PBS for 30 min at 37 °C. The cells were then incubated with an anti-LC3B antibody at a 1:500 dilution and an anti-LAMP1 antibody at a 1:200 dilution or an anti-SQSTM1/p62 antibody at a 1:1000 dilution and an anti-LAMP1 antibody at a 1:200 dilution for 1 h at 37 °C. After incubation for 1 h at 37 °C with the corresponding second antibody, samples were counterstained with 0.5* μ*g/ml 4’-,6-diamidino-2-phenylindole (DAPI) in PBS for 5 min, and the slides were mounted with FluorSave Reagent (Cat. #345789, Calbiochem, Billerica, MA, USA). Samples were observed with a laser-scanning confocal Nikon Eclipse Ti fluorescence microscope (Nikon Instruments, Inc., Melville, NY, USA).

### Image intensity profiling

Intensity profiling was performed with the NIS-Elements 4.5 Software (Nikon Instruments Inc., Melville, NY, USA) to estimate the overlapping rate between two fluorescence signals. A random line was drawn and its intensity profile was analyzed (Supplementary Figure S5). The peaks with fluorescence intensity equal to or stronger than 500 were considered as significant. The peaks with both fluorescence intensity equal to or stronger than 500 were considered as overlapping peaks. In the LC3B and LAMP1 co-staining experiments, the percentage of overlapping signals was defined by (the number of overlapping peaks)/(total green fluorescence peaks). In the experiments with mCherry-GFP-LC3B construct, the percentage of overlapping was defined by (the number of overlapping peaks)/(total red fluorescence peaks). We analyzed 10 profiles for each image. Statistical analysis was performed in Prism using one-way ANOVA with Dunnets *post-hoc* test (GraphPad Software, Inc., La Jolla, CA, USA).

### LysoTracker staining and magic red staining

Cells in the coverslips were stained with 50 nM LysoTracker Red DND-99 by directly adding this dye in the culture media, incubated for 30 min in a 5% CO_2_ atmosphere at 37 °C, and observed with the Nikon Eclipse Ti fluorescence microscope. Magic Red cathepsin B staining was performed according to the instructions of the manufacturer (Invitrogen). Cells were stained with Magic Red cathepsin B substrate for 1 h and observed with the Nikon Eclipse Ti fluorescence microscope.

### Quantitative reverse transcription real-time PCR

Total RNA was isolated with TRI reagent (Sigma, cat. #T9424). Reverse transcription was performed with total RNA using Maxima H Minus First Strand cDNA Synthesis Kit (SK1652, Thermo Fisher Scientific). Quantitative real-time PCR (qPCR) analysis was performed on an Eppendorf Master RealPlex real-time thermal cycler (Hauppauge, NY, USA) using the KAPA SYBR FAST qPCR Kits (#KK4602 Kapa Biosystems, Wilmington, MA, USA). The relative expression levels of the targeted genes were normalized to the that of the internal control 18 S rRNA gene. All reactions were run in triplicate. Human LC3B_F: 5′-CCGCACCTTCGAACAAAGAGT-3′ and LC3B_R: 5′-CACCCTTGTATCGTTCTATTATCACC-3′ were used as LC3B primers. Human SQSTM1/p62_F: 5′-GACCCACAGGGCTGAAGGAA-3′ and SQSTM1/p62_R: 5′-CCAGCCGCCTTCATCAGAGA-3′ were used as SQSTM1/p62 primers. Human 18 S rRNA_F: 5′-ATCAACTTTCGATGGTAGTCG-3′ and 18 S rRNA_R: 5′-TCCTTGGATGTGGTAGCCG-3′ were used as 18 S rRNA primers. Student’s *t*-test was used for statistical analysis.

### Western blotting

Western blotting was performed with minor modification as previously described.^6^ Briefly, cells were lysed in RIPA buffer supplemented with 1% SDS and proteinase inhibitor cocktail (1 : 100) (Sigma, P8340). Protein lysate of 10* μ*g for each sample was resolved by SDS-PAGE and transferred onto a PVDF membrane. After blocking with 5% skim milk, the membrane was probed with primary and secondary antibodies. The signal was developed with the Luminata Crescendo Western HRP substrate (#WBLUR0500, EMD Millipore, Billerica, MA, USA) and visualized with a UVP MultiSpectral Imaging System (UVP LLC, Upland, CA, USA).
