# Correction: Tenovin-6 impairs autophagy by inhibiting autophagic flux

**DOI:** 10.1038/s41419-018-0826-0

**Published:** 2018-07-16

**Authors:** Hongfeng Yuan, Brandon Tan, Shou-Jiang Gao

**Affiliations:** Department of Molecular Microbiology and Immunology, Keck School of Medicine, University of Southern California, Los Angeles, CA USA

Correction to: *Cell Death & Disease*; 10.1038/cddis.2017.25; published online 9 February 2017.

The PDF and HTML versions of the article have been updated to include the Creative Commons Attribution 4.0 International License information.

