# Supplementary material for: Tenovin-6 impairs autophagy by inhibiting autophagic flux
Source: Cell Death Dis. 2017 Feb 9;8(2):e2608–. doi: 10.1038/cddis.2017.25 (PMC5386474; doi:10.1038/cddis.2017.25)
Supplement: Supplementary Information [file cddis201725x1.docx]

**Tenovin-6 impairs autophagy by inhibiting autophagic flux**

Hongfeng Yuan^1^, Tan Brandon^1^ and Shou-Jiang Gao^1,^*

^1^Department of Molecular Microbiology and Immunology, Keck School of Medicine, University of Southern California, 1450 Biggy Street, Los Angeles, CA, USA

*Corresponding author: Shou-Jiang Gao, Department of Molecular Microbiology and Immunology, Keck School of Medicine, University of Southern California, 1450 Biggy Street, Los Angeles, CA, USA; Tel: +323 442 8028; Fax: +323 442 1721; E-mail: [shoujiag@usc.edu](mailto:shoujiag@usc.edu)

**
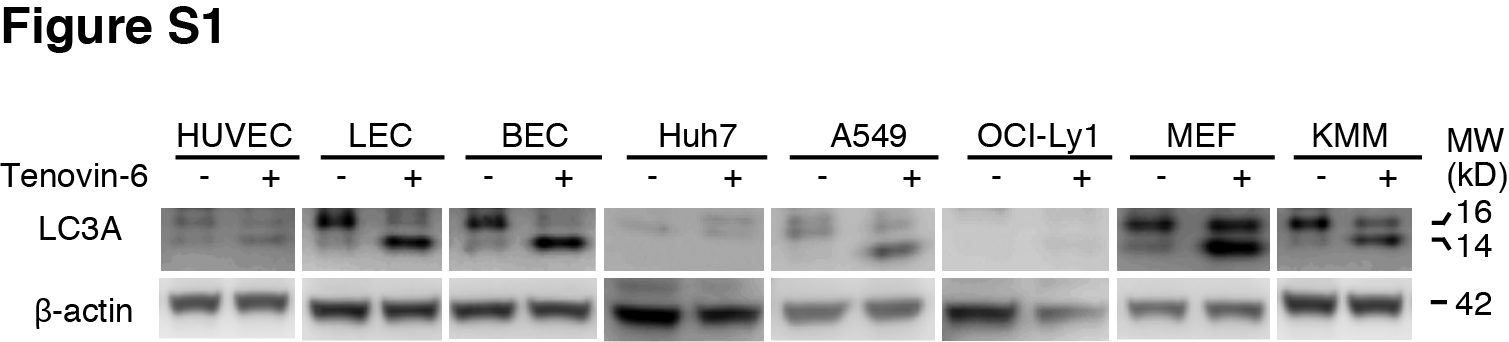
**

**Supplementary Figure S1.** LC3A and β-actin in a panel of different types of cells were examined by Western-blotting following tenovin-6 treatment for 24 h. Tenovin-6 was used at 5 μM except for A549 cells where 10 μM was used.


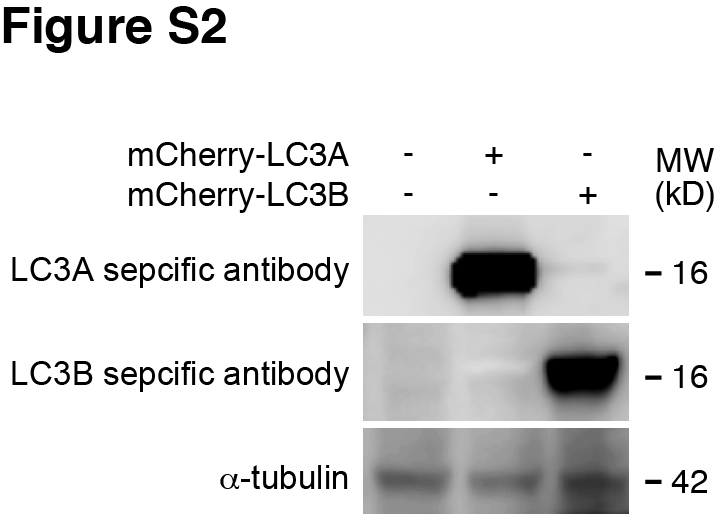


**Supplementary Figure S2.** Validation of the specificity of the antibody against LC3A or LC3B. The coding sequence of human LC3A or LC3B was in-frame cloned into the pCDH-mCherry-puromycin vector. The resulting vectors were then transfected into huh7 cells using lipofectamine 2000 transfection reagent. The cells were collected at 48 h post-transfection and examined by Western-blotting.


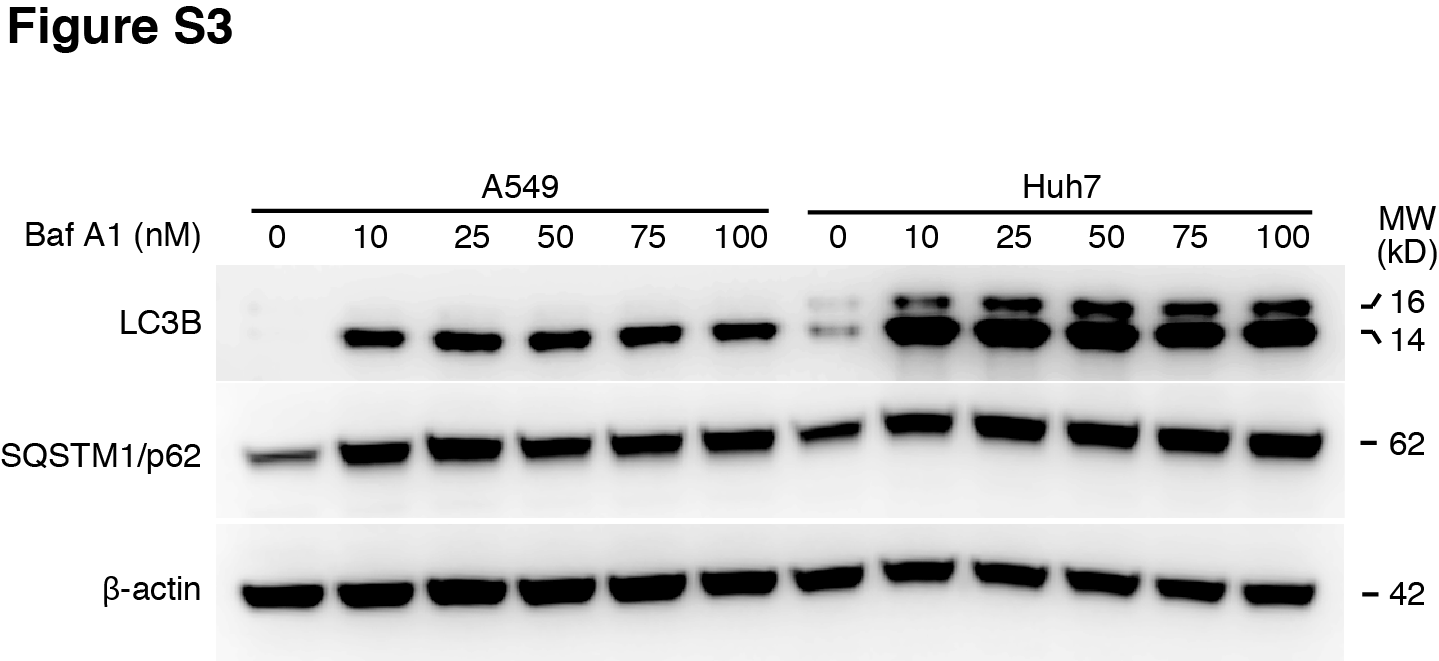


**Supplementary Figure S3.** LC3B, SQSTM1/p62 and β-actin were examined by Western-blotting in A549 and Huh7 cells following treatments with the indicated concentrations of bafilomycin A1 (Baf A1) for 8 h.

**
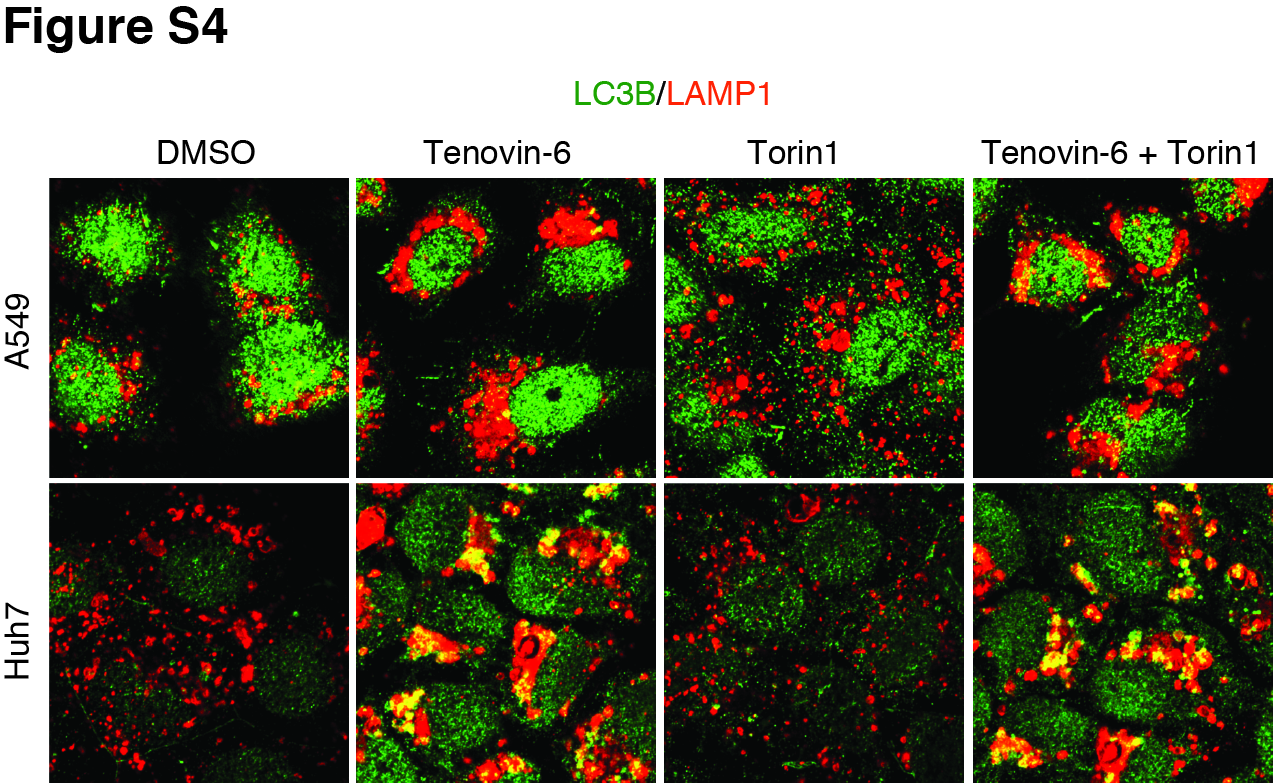
**

**Supplementary Figure S4**. Colocalization of LC3B with LAMP1 in A549 and Huh7 cells was examined by immunofluorescence assay following treatments with the indicated agents for 6 h. The concentrations of compounds used were 10 μM tenovin-6 and 250 nM Torin 1 for A549 cells; and 5 μM tenovin-6 and 250 nM Torin 1 for Huh7 cells.


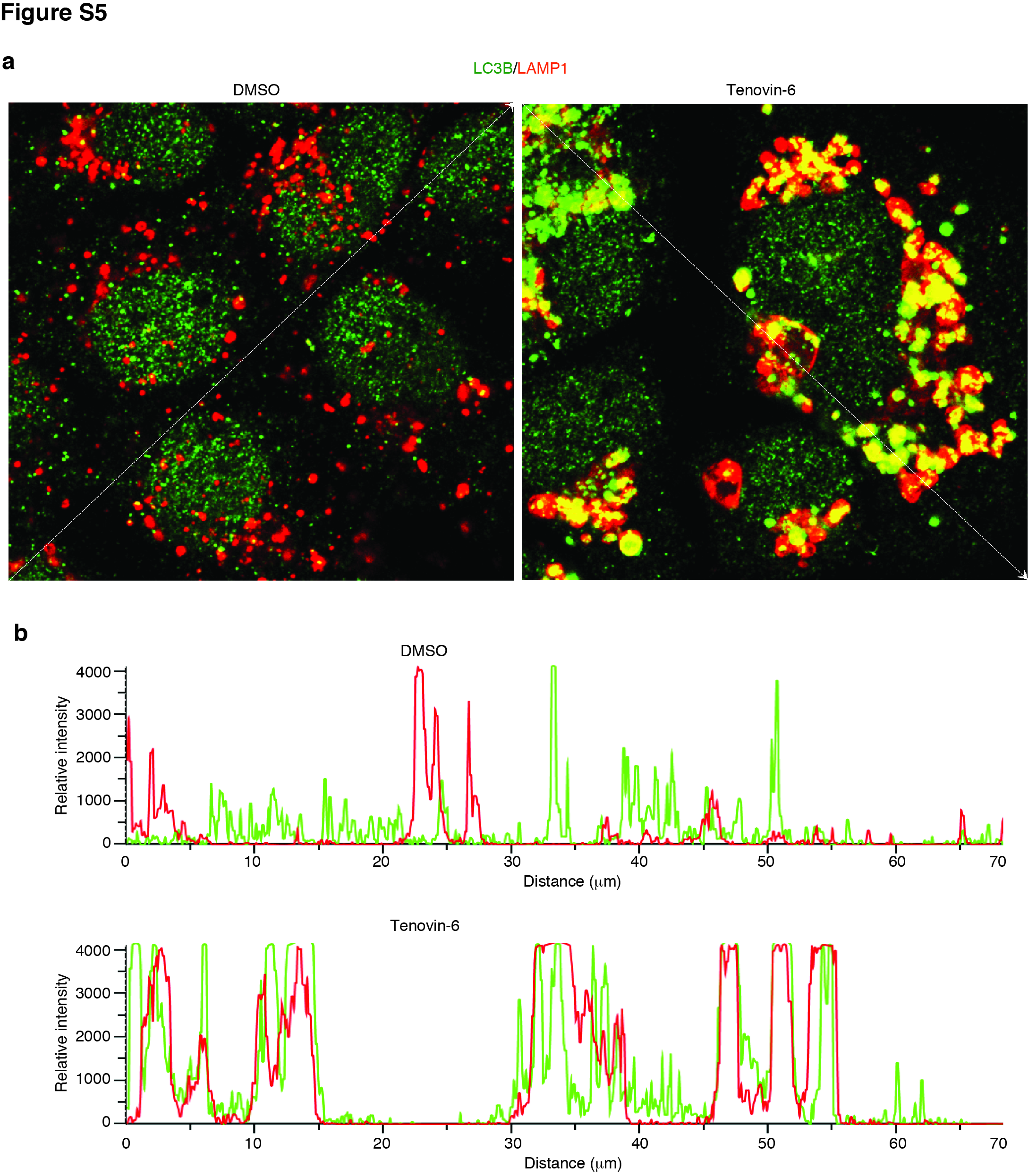


**Supplementary Figure S5**. Examples of fluorescence intensity profiling using the NIS element 4.5 software. A random line was drawn (a) and its intensity profile (b) was presented. The green signal was from LC3B and the red signal was from LAMP1. The peaks with both fluorescence signals equal to or stronger than 500 were considered as overlapping peaks. The images were obtained by immunofluorescence assay in Huh7 cells treated with DMSO or 5 μM tenovin-6 for 16 h.
